# Hsp90 Interacts With Tm-2^2^ and Is Essential for *Tm-2*^2^-Mediated Resistance to *Tobacco mosaic virus*

**DOI:** 10.3389/fpls.2018.00411

**Published:** 2018-04-10

**Authors:** Lichao Qian, Jinping Zhao, Yumei Du, Xijuan Zhao, Meng Han, Yule Liu

**Affiliations:** MOE Key Laboratory of Bioinformatics, Center for Plant Biology, Tsinghua-Peking Joint Center for Life Sciences, School of Life Sciences, Tsinghua University, Beijing, China

**Keywords:** *Tobacco mosaic virus*, plant-virus interaction, Hsp90, Tm-2^2^, SGT1, NBS-LRRs, *Nicotiana benthamiana*, immunity

## Abstract

The tomato resistance gene *Tm-2*^2^ encodes a coiled coil-nucleotide binding site-leucine rich repeat type resistance protein and confers effective immune response against tobamoviruses by detecting the presence of viral movement proteins (MPs). In this study, we show that the *Nicotiana benthamiana* Heat shock protein 90-kD (Hsp90) interacts with Tm-2^2^. Silencing of *Hsp90* reduced *Tm-2*^2^-mediated resistance to *Tobacco mosaic virus* (TMV) and the steady-state levels of Tm-2^2^ protein. Further, Hsp90 associates with SGT1 in yeast and in plant cells. These results suggest that Hsp90-SGT1 complex takes part in *Tm-2*^2^-mediated TMV resistance by functioning as chaperone to regulate Tm-2^2^ stability.

## Introduction

In the natural environment, pathogen microbes, such as viruses, bacteria, fungi, oomycetes, and nematodes, can cause disease in host plants. To counteract the pathogen attack, plants have evolved multilevel and sophisticated mechanisms to protect them from potential pathogen invasion. One of them is resistance (*R*) gene-mediated immunity (Dangl and Jones, 2001). *R* gene or its product recognizes the cognate pathogen avirulence protein directly or indirectly and activates powerful, specific resistance response. The activation of resistance pathways often culminate in a rapid hypersensitive response (HR) cell death at the pathogen infection sites. However, some *R* genes can induce resistance response without visible HR (Heath, 2000; Jones and Dangl, 2006). A well-known example is the *Rx* gene from potato, which mediates extreme resistance against *Potato virus X* (PVX) without any visible cell death at the initial infection sites (Bendahmane et al., 1999). Besides, tomato resistance gene *Tm-2*^2^ can mediate extreme resistance against tobamoviruses (Zhang et al., 2013). Nevertheless, they still have the potential to induce local cell death response in some conditions (Hall, 1980; Bendahmane et al., 1999; Du et al., 2013; Zhang et al., 2013).

Three tomato genes *Tm-1*, *Tm-2*, and *Tm-2*^2^ mediate resistance against tobamoviruses including *Tomato mosaic virus* (ToMV) and *Tobacco mosaic virus* (TMV). In contrast to *Tm-1* and *Tm-2*, *Tm-2*^2^ mediates much more durable resistance and has been applied in crop cultivation for several decades (Lanfermeijer et al., 2003). Tm-2^2^ contains a coiled-coil (CC) domain, a nucleotide binding site (NBS) domain and a leu-rich repeat (LRR) domain. Tm-2^2^ detects the presence of tobamovirus MPs (Weber and Pfitzner, 1998; Lanfermeijer et al., 2004) and functions on the plasma membrane (Chen et al., 2017). The Tyr-767 in Tm-2^2^ LRR domain is essential for the recognition of the MP of ToMV strain B7 (Kobayashi et al., 2011), suggesting that Tm-2^2^ recognizes viral MP through the LRR domain. In addition, Tm-2^2^ requires all domains for its activity and PM localization (Chen et al., 2017). The N-terminus of ToMV MP is important for Tm-2^2^ recognition (Weber et al., 2004; Chen et al., 2017). The expression of N-terminus (1-187aa) of viral MP is able to trigger *Tm-2*^2^-dependent cell death (Weber et al., 2004; Chen et al., 2017), although the two amino acid substitutions (S238R and K244E) in the C-terminus of ToMV MP lead to the overcoming of *Tm-2*^2^-mediated resistance (Weber et al., 1993). RuBisCO small subunit positively involves in *Tm-2*^2^-mediated extreme resistance (Zhao et al., 2013). Type I J-domain Hsp40 proteins (called NbMIP1s) and co-chaperone SGT1 are also indispensable for *Tm-2*^2^-mediated extreme resistance (Du et al., 2013; Zhao et al., 2013). Nevertheless, the molecular mechanism of *Tm-2*^2^-mediated virus resistance is largely unknown.

Heat shock protein 90-kD (Hsp90) is a molecular chaperone required for the stability and activity of many proteins during a variety of cellular processes, such as protein maturation, complex assembly, signal transduction and genetic buffering. For examples, plant Hsp90 can facilitate the folding of mammalian glucocorticoid receptors *in vitro* (Stancato et al., 1996). Hsp90 associates with the 26S proteasome and is critical for ATP-dependent assembly and maintenance of the 26S proteasome (Imai et al., 2003). Hsp90 is also essential for plant disease resistance. Hsp90 modulates *RPS2*- and *RPM1*-mediated resistance in *Arabidopsis* (Hubert et al., 2003; Takahashi et al., 2003). Silencing *Hsp90* using viral-induced gene silencing (VIGS) suppressed the plant resistance conferred by several *R* genes including *N*, *Rx* and *Pto* in *N. benthamiana* (Kanzaki et al., 2003; Lu et al., 2003; Liu et al., 2004). Suppression of *TaHsp90.2* or *TaHsp90.3* compromised the resistance against stripe rust fungus in common wheat (Wang et al., 2011). Knock down of *Hsp90* compromised *I-2* mediated cell death completely, suggesting that *Hsp90* is essential for the tomato *I-2*-mediated resistance (de la Fuente van Bentem et al., 2005). In addition, *Hsp90* is also involved in *Mi-1*-mediated pest immune response (Bhattarai et al., 2007). SGT1 interacts with Hsp90, and functions as a co-chaperone of Hsp90 to modulate the immune response by regulating R protein stability (Lu et al., 2003; Liu et al., 2004; Zhang et al., 2004).

In this study, we report that *N. benthamiana* Hsp90 associates with Tm-2^2^ *in vitro* and *in vivo*, and plays an essential role in *Tm-2*^2^-mediated TMV resistance by regulating its protein stability.

## Materials and Methods

### Plant Materials, Plasmids and Pathogens

Wild type *N. benthamiana* and transgenic *Tm-2*^2^ *N. benthamiana* plants were described (Zhang et al., 2013). All *N. benthamiana* plants were grown in greenhouse at 23–25°C under a 16 h light/8 h dark cycle with 40–60% relative humidity and 40 umol m^-2^ s^-1^ white light illumination.

DNA fragments of Tm-2^2^-nLUC, cLUC-NbHsp90, Tm-2^2^-4 × myc and 3 × HA-NbHsp90 were generated by overlapping PCR, and then cloned into T-DNA vector pJG045, a pCAMBIA1300-based T-DNA vector (Zhao et al., 2013). PVX-based vector PVX-LIC was described (Zhao et al., 2016). The coding sequences of *NbHsp90* (AY368904: nt1859-2103) was RT-PCR amplified and cloned into PVX-LIC vector for VIGS. All constructs were verified by DNA sequencing.

GFP-tagged TMV (TMV-GFP) was described (Liu et al., 2002a).

### Yeast Two-Hybrid Assays

The full-length *Tm-2*^2^, Tm-2^2^-LRR were PCR amplified and cloned into the LexA DNA binding domain (BD) containing yeast vector pYL302 (Liu et al., 2002b) to generate the bait vectors BD-Tm-2^2^ and BD-Tm-2^2^-LRR. The full-length NbHsp90 cDNA was amplified by RT-PCR and cloned into the B42 activation domain (AD)-containing yeast vector pJG4-5 NbHsp90 to generate AD-NbHsp90. The yeast two-hybrid prey library containing tomato cDNAs (Liu et al., 2002b) was used to screen Tm-2^2^-LRR-binding proteins. The yeast two-hybrid screening and interaction assay were performed as described (Liu et al., 2002b).

### Luciferase Complementation Imaging (LCI) Assays

Luciferase complementation imaging assay was conducted as described (Chen et al., 2008; Du et al., 2013). All tested combinations were agroinfiltrated into leaves of 4 weeks old *N. benthamiana*. The leaves were collected 48 h post infiltration (hpi) and sprayed with luciferin (1 mM) followed by capturing the LUC image using a cooled CCD imaging apparatus (iXon, Andor Technology).

### Protein Analysis and Co-Immunoprecipitation (Co-IP)

We used *Agrobacterium*-mediated transient expression approach for protein expression. The GV2260 strains containing the relevant expression vector were infiltrated into leaves of *N. benthamiana*. The leaves were collected at 48 hpi for protein extraction. Protein samples were extracted with Laemmli buffer (Laemmli, 1970) and subjected to electrophoresis on SDS–PAGE gel followed by western blot assays using anti-myc (Abmart) or anti-Hsp90 (Santa Cruz Biotechnology) primary antibodies and were detected using Pierce ECL western blotting substrate (Pierce).

For Co-IP assays, HA-NbHsp90 was co-expressed with Tm2^2^-myc or cLUC-myc control in *N. benthamiana*. The infiltrated leaf tissues were collected 48 hpi and total protein extracts were subjected to IP procedure using anti-HA beads under agitation at 4°C for 2 h, then the beads were washed four times with ice-cold extraction buffer at 4°C (Du et al., 2013). The immunoprecipitates and input were extracted with Laemmli buffer and subjected to electrophoresis on SDS–PAGE gel followed by western blot assays using anti-myc or anti-HA antibody (Cell Signaling Technology) and detected using Pierce ECL western blotting substrate (Pierce).

### Gene Expression Assays

RT-PCR and quantitative RT-PCR were conducted, respectively, as described (Liu et al., 2002a; Wang et al., 2013). *NbActin* mRNA was served as an internal control for normalization. Primers were designed with Primer3web^1^.

### VIGS, Virus Inoculation and GFP Imaging

For VIGS assays, PVX: NbHsp90 or control plasmid was transformed into *Agrobacterium tumefaciens* strains GV2260 and then infiltrated into the leaves of 4 weeks old *N. benthamiana* plants. For TMV infection, TMV-GFP was agroinfiltrated into the plant leaves (Liu et al., 2002a). Each silencing experiment was repeated using at least five independent plants at least four times Pictures were photographed under white and UV light using a Canon 650D camera.

## Results

### Identification of NbHsp90 as Tm-2^2^-Interacting Partner

Tm-2^2^ LRR domain is reported to be involved in virus recognition (Kobayashi et al., 2011). To understand Tm-2^2^ action, we conducted a yeast two-hybrid screen of a tomato cDNA library using Tm-2^2^-LRR (aa: 444-961) as bait, and identified several host proteins interacted with Tm-2^2^ (Liu et al., 2004; Du et al., 2013). In this screen, we identified SGT1 and NbMIP1s as partners interacting with Tm-2^2^ (Liu et al., 2002b; Du et al., 2013). In addition, Hsp90 (AY368906) (Liu et al., 2004) was also identified to interact with Tm-2^2^. Further, two *N. benthamiana* Hsp90 homologs (AY368904, AY368905) (Wang et al., 2011) were identified to share high identity with tomato Hsp90 (AY368906). It should be noted that two *NbHsp90* homologs are almost identical to one another. Because *N. benthamiana* is an allotetraploid, we believe that these two *NbHsp90* homologs are two alleles of same gene.

### NbHsp90 Interacts With Tm-2^2^ in Yeast

**FIGURE 1:**
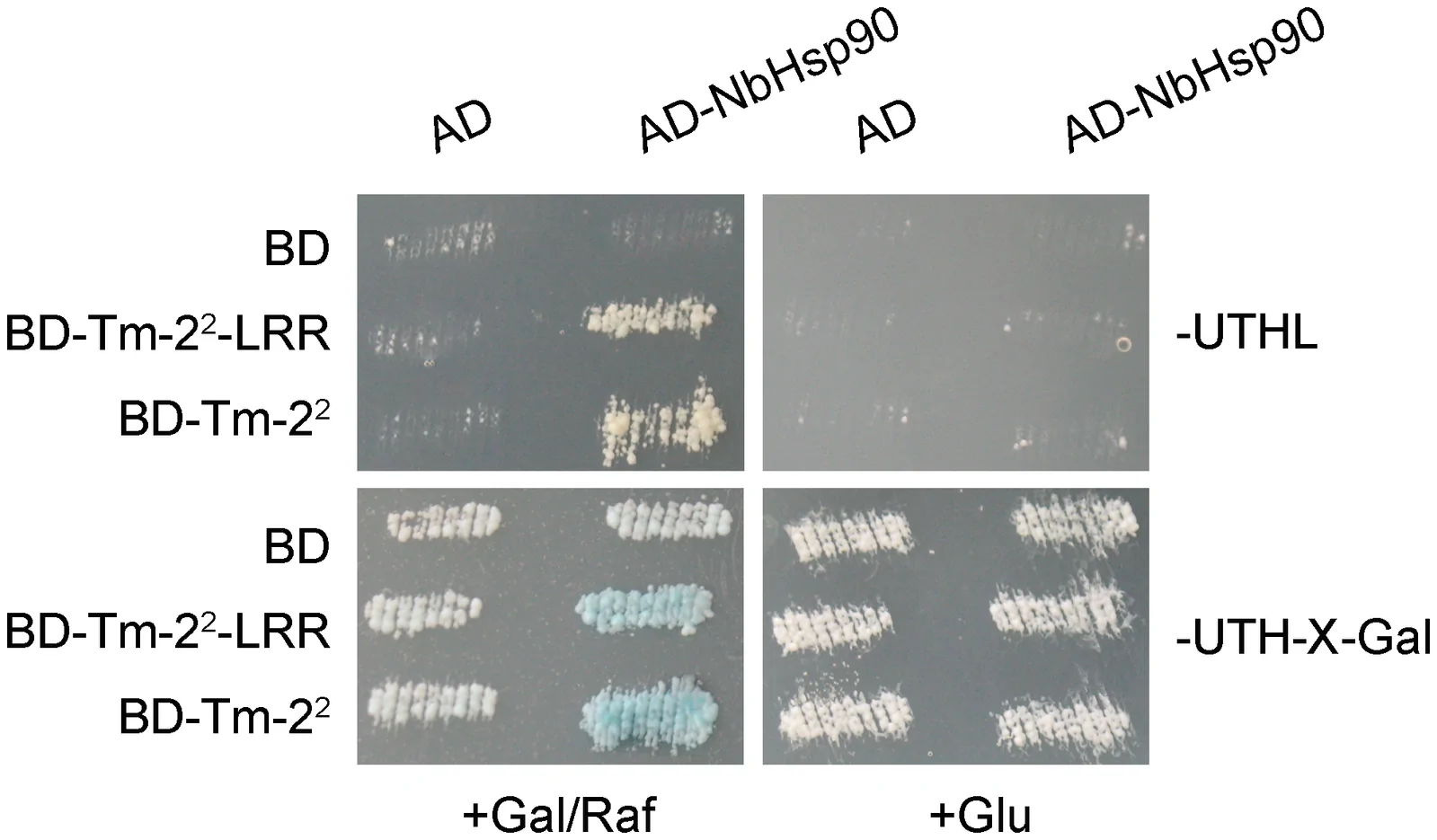
NbHsp90 Interacts with Tm-2^2^ in Yeast. Yeast cells containing NLS-LexA BD-Tm-2^2^ or BD-Tm-2^2^-LRR baits transformed with AD-NbHsp90 grew on Leucine deficient medium (Leu^-^) and turned blue on X-gal medium containing galactose (Gal) and raffinose (Raf) but not on medium containing glucose (Glu) at 28°C for 4 days. Yeast cells transformed with either AD or BD empty vector alone were used as negative control.

Further, we verified the interaction of NbHsp90 with Tm-2^2^ using LexA based yeast two-hybrid system (Du et al., 2013). Both BD- and AD- fusion genes were driven by a galactose-inducible promoter. Yeasts transformed AD-NbHsp90 and BD-Tm-2^2^ or BD-Tm-2^2^-LRR grew on galactose medium lacking leucine, and became blue on medium containing X-gal and galactose/raffinose but not glucose (**Figure 1**). In contrast, control yeasts containing AD or BD alone did not grow on the medium lacking leucine or turn blue on X-gal medium (**Figure 1**). Therefore, both Tm-2^2^ and Tm-2^2^-LRR interact with NbHsp90 in yeast.

### NbHsp90 Interacts With Tm-2^2^ in Plant Cells

**FIGURE 2:**
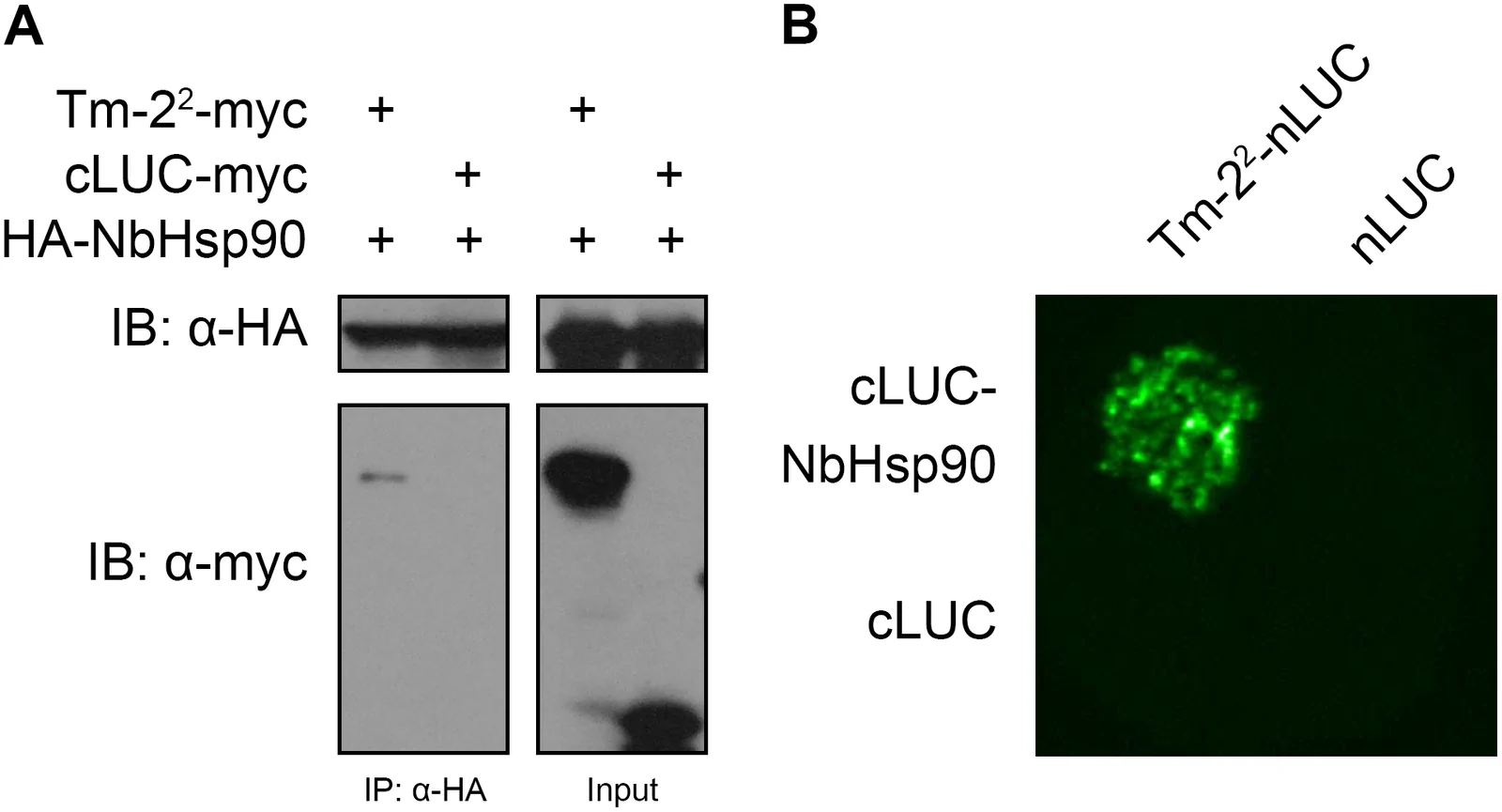
NbHsp90 Interacts with Tm-2^2^ in Plant Cells. **(A)** NbHsp90 co-immunoprecipitated (Co-IP) with Tm-2^2^. HA-NbHsp90 was transiently expressed with Tm-2^2^-myc in *Nicotiana benthamiana* leaves by agroinfiltration. HA-NbHsp90 co-expressed with cLUC-myc was used as negative control. At 48 h post infiltration (hpi), total protein extracts were immunoprecipitated (IP) with anti-HA beads and the resulting precipitates were assessed by western blotting using anti-HA antibodies (upper panel, left) and anti-myc antibodies (lower panel, left). The presence of HA-NbHsp90, Tm-2^2^-myc and cLUC-myc in the immunoprecipitates and cell lysates were also analyzed (right). IB, Immunoblot. **(B)** Firefly luciferase complementation imaging (LCI) assays for the *in vivo* interaction of NbHsp90 with Tm-2^2^. cLUC-NbHsp90 was transiently co-expressed with Tm-2^2^-nLUC or nLUC in *N. benthamiana* leaves followed by LCI assay.

To examine whether NbHsp90 interacts with Tm-2^2^ in plant cells, we conducted Co-IP assay. The HA-tagged NbHsp90 (HA-NbHsp90) was co-expressed with myc-tagged Tm-2^2^ (Tm-2^2^-myc) or cLUC-myc (as a negative control) in *N. benthamiana* leaves. Leaf tissues were detached 48 hpi. Total protein was extracted and immunoprecipitated using anti-HA agarose, followed by western blot assays with anti-HA and anti-myc antibodies. We found that NbHsp90 co-immunoprecipitated with Tm-2^2^, but not with cLUC-myc (**Figure 2A**).

We further validated the *in vivo* interaction of NbHsp90 with Tm-2^2^ via LCI assay (Chen et al., 2008). N-terminus (nLUC) and C-terminus (cLUC) of the firefly luciferase can reconstitute active enzyme when they are fused, respectively, with two interacting proteins. To this end, we generated Tm-2^2^-nLUC and cLUC-NbHsp90 and co-expressed them in *N. benthamiana* leaves. Positive signals were observed for the combination of cLUC-NbHsp90 with Tm-2^2^-nLUC (**Figure 2B**). However, no signals were observed for the control combinations (cLUC-NbHsp90 plus nLUC, cLUC plus Tm-2^2^-nLUC) (**Figure 2B**). These results, along with our Co-IP data, suggest that NbHsp90 interacts with Tm-2^2^ in plant cells.

### *NbHsp90* Is Essential for *Tm-2^*2*^*-Mediated TMV Resistance

**FIGURE 3:**
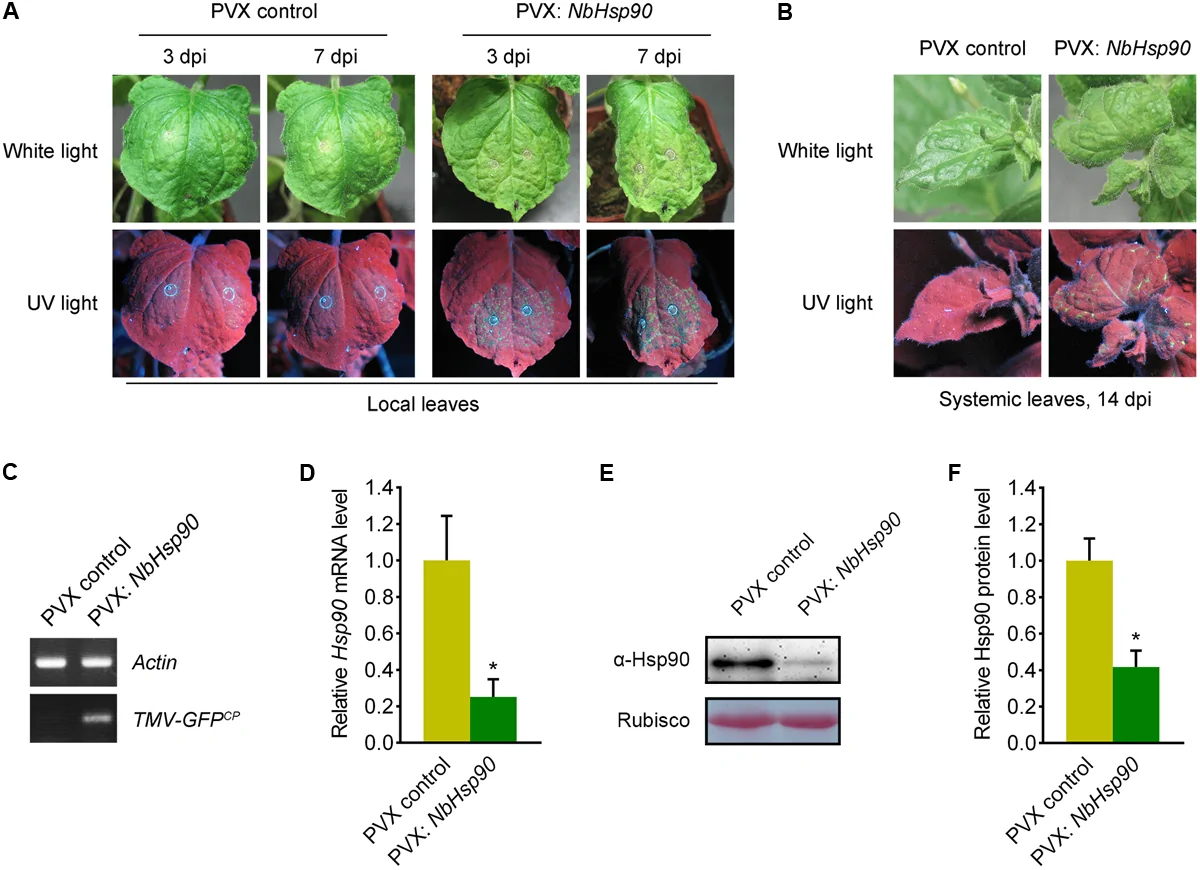
Silencing of *NbHsp90* Compromised *Tm-2*^2^-mediated Resistance to TMV. **(A)** Silencing of *NbHsp90* caused the appearance of TMV-GFP infection foci and visible HR lesions (right) in the inoculated leaves of *NbHsp90*-silenced *Tm-2*^2^-containing plants. PVX-infected *Tm-2*^2^-containing plants were used as negative controls (left). **(B)** TMV-GFP spread into the systemic leaves of *NbHsp90*-silenced *Tm-2*^2^-containing plants (right) but not the PVX control plants (left). Photos were taken at 14 days post TMV-GFP infection (dpi). **(C)** RT-PCR to confirm the presence of TMV-GFP in systemic leaves of *NbHsp90*-silenced *Tm-2*^2^-containing plants (right) but not the PVX control plants (left). **(D)** Quantitative RT-PCR assays to confirm the reduction in *NbHsp90* mRNA (means ± SEM, *n* = 3). ^∗^*P* < 0.05, Student’s *t*-test. *NbActin* mRNA levels were used as the internal control. **(E,F)** Western blot assays to confirm the reduction in NbHsp90 protein level (means ± SEM, *n* = 4). ^∗^*P* < 0.05, Student’s *t*-test. Equal loading of protein samples was validated by Ponceau Red staining of Rubisco subunit.

To determine the role of NbHsp90 in *N. benthamiana* plants, we cloned a partial fragment of *NbHsp90* (nt: 1859-2103) into PVX VIGS vector PVX-LIC (Zhao et al., 2016) to generate PVX-NbHsp90, and the PVX vector alone was used as negative control. Silencing of *NbHsp90* induced developmental abnormalities including stopping growing and severely stunted (Supplementary Figure S1) (Liu et al., 2004), and quantitative RT-PCR data showed that the *NbHsp90* mRNA level was greatly reduced in the PVX-NbHsp90 plants compared to the PVX vector plants (**Figure 3D**). Further, western blot assays using Hsp90-specific antibody showed that silencing of *NbHsp90* greatly reduced Hsp90 protein level (**Figures 3E,F**).

Then we investigated the role of *NbHsp90* in *Tm-2*^2^-mediated TMV resistance. To this end, we performed this experiment in transgenic *Tm-2*^2^ *N. benthamiana* plants (thereafter called *Tm-2*^2^ plants) that show effective resistance against TMV-GFP (Zhang et al., 2013). We agroinfiltrated the *NbHsp90*-silenced and PVX control non-silenced *Tm-2*^2^ plants with *Agrobacterium* containing TMV-GFP plasmid (Liu et al., 2002a) and observed virus infection foci in inoculated leaves at 3 dpi (**Figure 3A**, left). Compared to the non-silenced *Tm-2*^2^ plants, *NbHsp90*-silenced *Tm-2*^2^ plants developed more TMV-GFP foci and subsequently developed obvious necrosis lesions at 7 dpi in inoculated leaves (**Figure 3A**, right). Furthermore, at 14 dpi TMV-GFP spread into the systemic leaves of *NbHsp90*-silenced *Tm-2*^2^ plants but not that of non-silenced control *Tm-2*^2^ plants (**Figure 3B**). RT-PCR showed that TMV RNA was readily detected in the systemic leaves of *NbHsp90*-silenced *Tm-2*^2^ plants but not in the systemic leaves of non-silenced *Tm-2*^2^ plants (**Figure 3C**). Taken together, these findings suggest that *Tm-2*^2^-mediated TMV resistance requires *NbHsp90*.

### NbHsp90 Is Essential for Stability of Tm-2^2^ Protein

**FIGURE 4:**
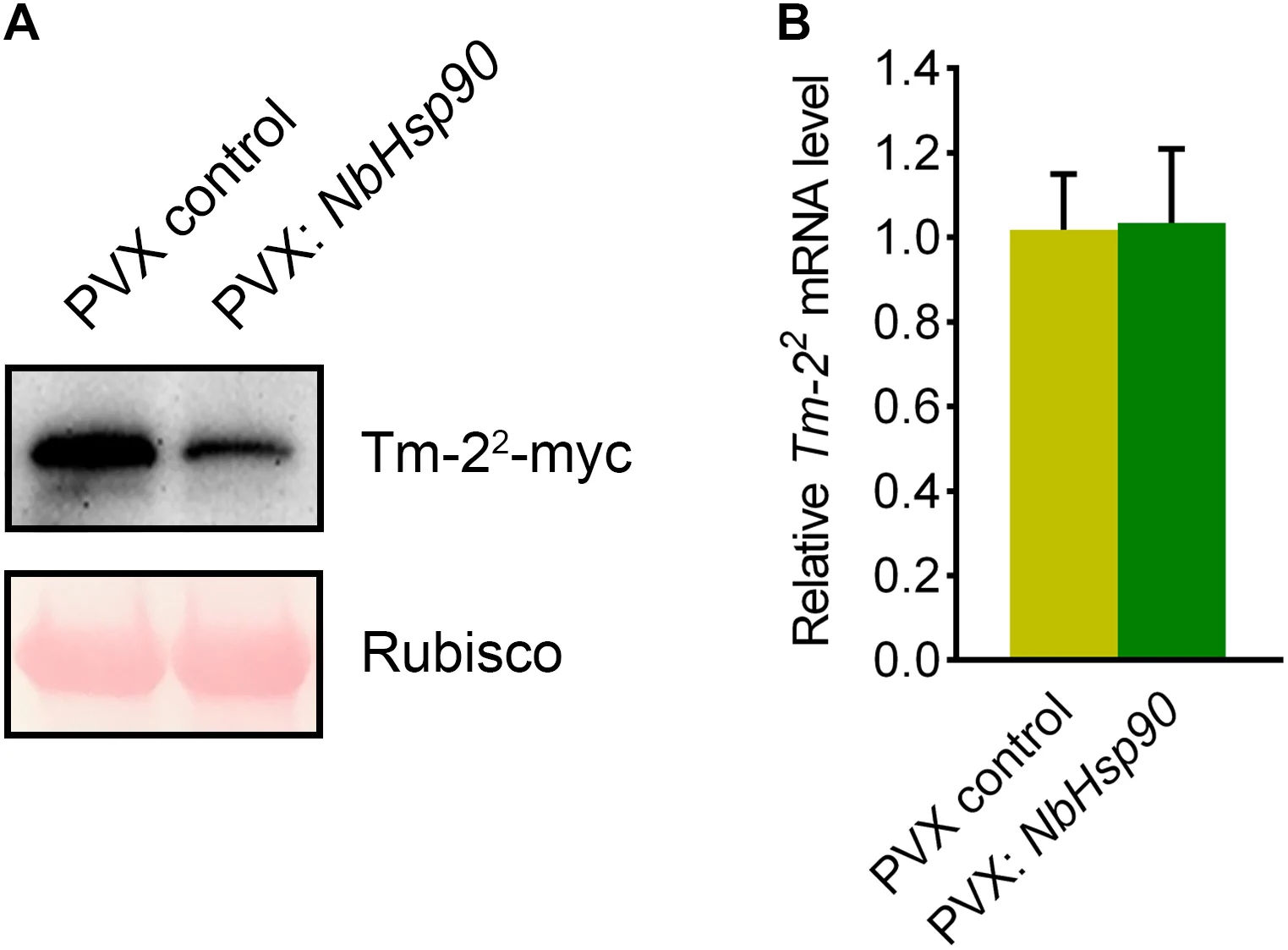
NbHsp90 is Required for Tm-2^2^ Protein Stability. **(A)** Tm-2^2^-myc was transiently agroinfiltrated into leaves of *NbHsp90* silenced plants and control plants, respectively, proteins were extracted and analyzed by SDS–PAGE, followed by western blot using anti-myc antibody (upper panels). Equal loading of protein samples was validated by Ponceau Red staining of Rubisco subunit (lower panels). **(B)** Real-time RT-PCR assays showed that silencing of *NbHsp90* did not affect *Tm-2*^2^ mRNA level (means ± SEM, *n* = 5). *NbActin* mRNA levels were used as the internal control.

NbHsp90 is essential for *Rx*-mediated PVX resistance by regulating the protein level of Rx-4 × HA in *N. benthamiana* (Lu et al., 2003). RPM1 level is also reduced in *Arabidopsis hsp90.2* mutant (Hubert et al., 2003). To investigate how NbHsp90 regulates *Tm-2*^2^-mediated TMV resistance, we expressed Tm-2^2^-myc in *NbHsp90*-silenced and non-silenced *N. benthamiana* plants to investigate the effect of *NbHsp90* silencing on Tm-2^2^ protein accumulation (Du et al., 2013). Western blot assays showed that *NbHsp90*-silenced plants accumulated less Tm-2^2^ protein compared with non-silenced control plants (**Figure 4A**). However, quantitative RT-PCR assay indicated that *NbHsp90* silencing had no significant effect on *Tm-2*^2^ mRNA level (**Figure 4B**). Taken together, these findings indicate that NbHsp90 is indispensable for Tm-2^2^ protein stability.

### NbHsp90 Interacts With NbSGT1 in Yeast and in Plant Cells

**FIGURE 5:**
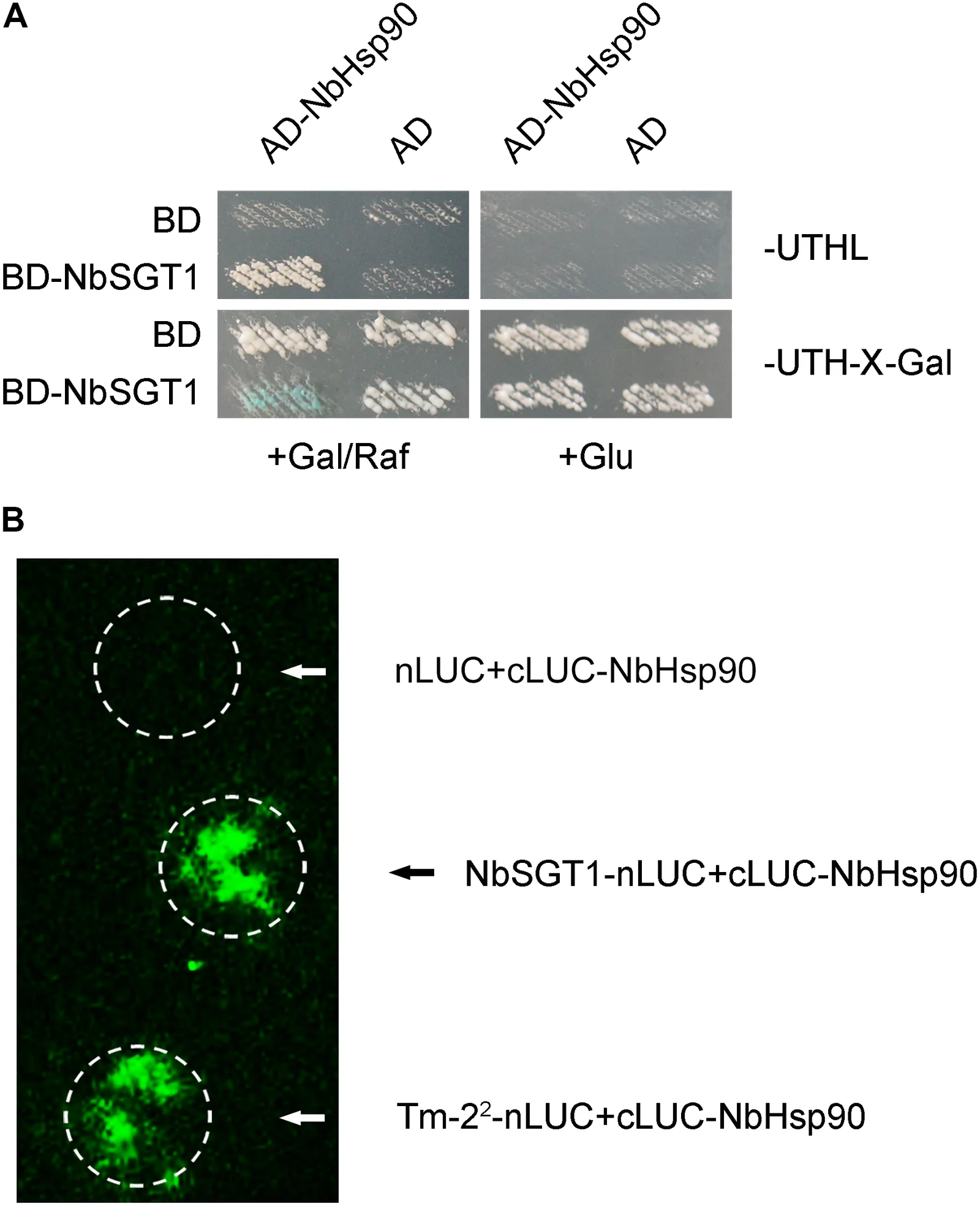
NbHsp90 Interacts with NbSGT1 in Yeast and in Plant Cells. **(A)** Yeast cells harboring NLS-LexA BD-SGT1 baits transformed with AD-NbHsp90 grew on Leu^-^ deficient medium and turned blue on X-gal medium containing galactose (Gal) and raffinose (Raf) but not on medium containing glucose (Glu) at 28°C for 4 days. Yeast cells transformed with either AD or BD empty vector alone were used as negative control. **(B)** Firefly LCI assays for the *in vivo* interaction between NbHsp90 with SGT1. cLUC-NbHsp90 was transiently co-expressed with SGT1-nLUC or nLUC in *N. benthamiana* leaves followed by LCI assay.

We had reported that NbSGT1 interacts with Tm-2^2^ and is essential for *Tm-2*^2^-mediated TMV resistance by regulating Tm-2^2^ protein stability (Du et al., 2013). To investigate whether Hsp90 regulates Tm-2^2^ protein stability through NbHsp90-NbSGT1 chaperone complex, we first tested the interaction between NbHsp90 and NbSGT1 using yeast two-hybrid system. Yeast cells harboring both AD-NbHsp90 and BD-SGT1 grew on medium lacking leucine, and became blue on medium containing X-gal and galactose/raffinose but not glucose (**Figure 5A**). However, control yeasts containing AD or BD vector alone neither grew on the medium lacking leucine nor turned blue on X-gal medium (**Figure 5A**). NbHsp90 therefore interacts with NbSGT1 in yeast. Further, we performed LCI assays to investigate whether NbHsp90 interacts with NbSGT1 in plant cells. We found that cLUC-NbHsp90 interacts with NbSGT1-nLUC, but not with empty cLUC control (**Figure 5B**). These experiments show that NbHsp90 interacts with NbSGT1 in both yeast and plant.

## Discussion

### The Role of Hsp90 in Plant *R* Gene-Mediated Resistance Against Viruses

Hsp90 is a highly abundant and conserved cellular chaperone known to regulate various biological processes, and is reported to play crucial roles in plant disease resistance (Hubert et al., 2003; Lu et al., 2003; Takahashi et al., 2003; Liu et al., 2004). Using high throughput VIGS assay, Hsp90 was characterized to be a cofactor of Rx protein to stabilize its protein level (Lu et al., 2003). Association of Hsp90 with NBS-LRR proteins has been reported (Hubert et al., 2003; Liu et al., 2004; Zhu et al., 2017). Hsp90 associates with N protein directly to modulate the immune response to TMV (Liu et al., 2004). Hsp90, SGT1 and Rar1 form a complex and act as co-chaperones during virus disease resistance (Picard, 2002). In fact, the structurally conserved Hsp90-SGT1 complex (Seo et al., 2008; Shirasu, 2009) are functionally required for different NBS-LRR proteins’ function as immune modulator against various pathogens including bacteria (Takahashi et al., 2003; Zhang et al., 2004), fungi (Bieri et al., 2004; Thao et al., 2007), oomycetes (Michael Weaver et al., 2006; Bhaskar et al., 2008; Oh et al., 2014), nematodes (Bhattarai et al., 2007; Zhu et al., 2017). Here we reported that Hsp90 directly interacts with Tm-2^2^, a CC-NBS-LRR type of resistance protein, confers robust immune response against tobamoviruses. Besides, we found that Hsp90 interacts with SGT1 in yeast and in plant cells. This finding is consistent with our earlier report that SGT1 participates in *Tm-2*^2^-mediated resistance against TMV by regulating protein stability of Tm-2^2^ through its interaction with Tm-2^2^ (Du et al., 2013). Hsp90 and its co-chaperone SGT1 may facilitate the folding and maturation of R proteins. The misfolded R proteins can be eliminated by protein quality control machine. In such a scenario, knock down of *Hsp90* or *SGT1* decreases the amount of R protein and compromises R protein function (Lu et al., 2003; Liu et al., 2004; Zhang et al., 2004). Accordingly, silencing of *Hsp90* suppressed *Tm-2*^2^-mediated TMV resistance and reduced the stability of Tm-2^2^ protein. Taken together, our findings further support that Hsp90-SGT1 chaperone mediates the stabilization and maturation of R proteins.

### The Role of Hsp90-Related Co-chaperones in Plant-Virus Interaction

The DnaJ/Hsp40 works as a co-chaperone in Hsp90-Hsp70-Hsp40 complex, and can also form complex with Hsp90 during protein folding process (Verchot, 2012). DnaJ/Hsp40 proteins play dual roles in plant virus infection and host resistance. Via directly interaction with virus effectors, varied DnaJ/Hsp40 type proteins positively or negatively affect the replication and/or movement of several plant viruses including PVX, PVY, TSWV, and TMV (Soellick et al., 2000; Hofius et al., 2007; Cho et al., 2012; Shimizu et al., 2009). In addition, type I DnaJ/Hsp40 protein NbMIP1s also interact with Tm-2^2^ and SGT1 and are required for *Tm-2*^2^-mediated resistance by sustaining the protein stability (Du et al., 2013). Type-III DnaJ/Hsp40 plays a positive role in plant defense against *Soybean mosaic virus* in soybean (Liu and Whitham, 2013).

In this study, we found that Hsp90, like NbMIP1, is required for *Tm-2*^2^-mediated resistance against TMV. However, no interaction between TMV MP and Hsp90 is detected (data not shown). In addition, *NbHsp90* expression (at mRNA and protein levels) was not induced by TMV infection and *Tm-2*^2^-mediate resistance (Supplementary Figure S2). Considered that NbMIP1 interacts with TMV MP, and *NbMIP1s* is induced by TMV infection and *Tm-2*^2^-mediate resistance (Du et al., 2013), Hsp90 and NbMIP1s may exist in different cellular protein complexes during plant-virus interaction (Cintron and Toft, 2006). Indeed, Hsp90 and DnaJ/HsP40 proteins are not necessarily linked in their role as chaperones to facilitate the folding of diverse client proteins during different biological processes such as virus infection and plant resistance (Li et al., 2012; Verchot, 2012), and the chaperone machinery Hsp90-Sgt1 and Hsp90-Hsp40 is of different partnership for client recruitment and folding (Park and Seo, 2015).

## Author Contributions

YL, JZ, LQ, and YD designed the experiments, analyzed the data, and prepared the manuscript. JZ, LQ, YD, XZ, and MH carried out the experiments. All authors contributed the revision of manuscript through the discussion.

## Conflict of Interest Statement

The authors declare that the research was conducted in the absence of any commercial or financial relationships that could be construed as a potential conflict of interest.

## Footnotes

1
http://primer3.ut.ee/
