# Correction: Hsp90 interacts with Tm-22 and is essential for Tm-22-mediated resistance to *Tobacco mosaic virus*

**DOI:** 10.3389/fpls.2026.1934563

**Published:** 2026-08-14

**Authors:** Lichao Qian, Jinping Zhao, Yumei Du, Xijuan Zhao, Meng Han, Yule Liu

**Affiliations:** MOE Key Laboratory of Bioinformatics, Center for Plant Biology, Tsinghua-Peking Joint Center for Life Sciences, School of Life Sciences, Tsinghua University, Beijing, China

**Keywords:** *Tobacco mosaic virus*, plant-virus interaction, Hsp90, Tm-2^2^, SGT1, NBS-LRRs, immunity

Author Jinping Zhao was erroneously assigned to affiliation “Texas A&M AgriLife Research and Extension Center at Dallas, Texas A&M University, Dallas, TX, United States”. This affiliation has now been removed for author Jinping Zhao.

The original version of this article has been updated.

