# Supplementary material for: Hsp90 Interacts With Tm-22 and Is Essential for Tm-22-Mediated Resistance to Tobacco mosaic virus
Source: Front Plant Sci. 2018 Apr 10;9:411. doi: 10.3389/fpls.2018.00411 (PMC5902563; doi:10.3389/fpls.2018.00411)
Supplement: Supplementary file 1 [file Data_Sheet_1.docx]

Supplementary Material

**Hsp90 interacts with Tm-2^2^** **and is essential for *Tm-2^2^*-mediated resistance to *Tobacco mosaic virus***

# Lichao Qian^1,#^, Jinping Zhao^1,2,#,*^, Yumei Du^1^, Xijuan Zhao^1^, Meng Han^1^ and Yule Liu^1,*^

1. MOE Key Laboratory of Bioinformatics, Center for Plant Biology, Tsinghua-Peking Joint Center for Life Sciences, School of Life Sciences, Tsinghua University, Beijing 100084, China
2. Texas A&M AgriLife Research Center at Dallas, Texas A&M University System, Dallas, TX, 75252, USA

# These authors have contributed equally to this work.

# * Corresponding:

Yule Liu

[yuleliu@mail.tsinghua.edu.cn](mailto:yuleliu@mail.tsinghua.edu.cn)

Jinping Zhao

[jinpingzhao@sina.c](mailto:jinpingzhao@sina.com)n

The Supplementary Material for this article includes 2 figures.


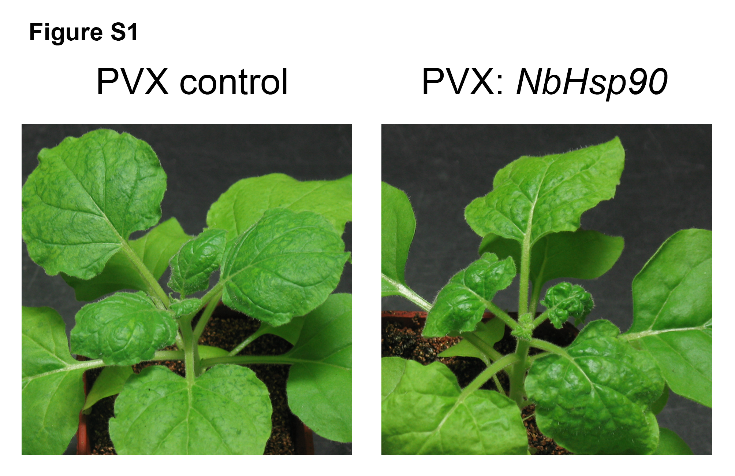


**Supplement Figure 1. Silencing of *NbHsp90* induced developmental abnormalities.** Silencing of *NbHsp90* using PVX-NbHsp90 induced developmental abnormalities including stopping growing and severely stunted compared to PVX control plants.


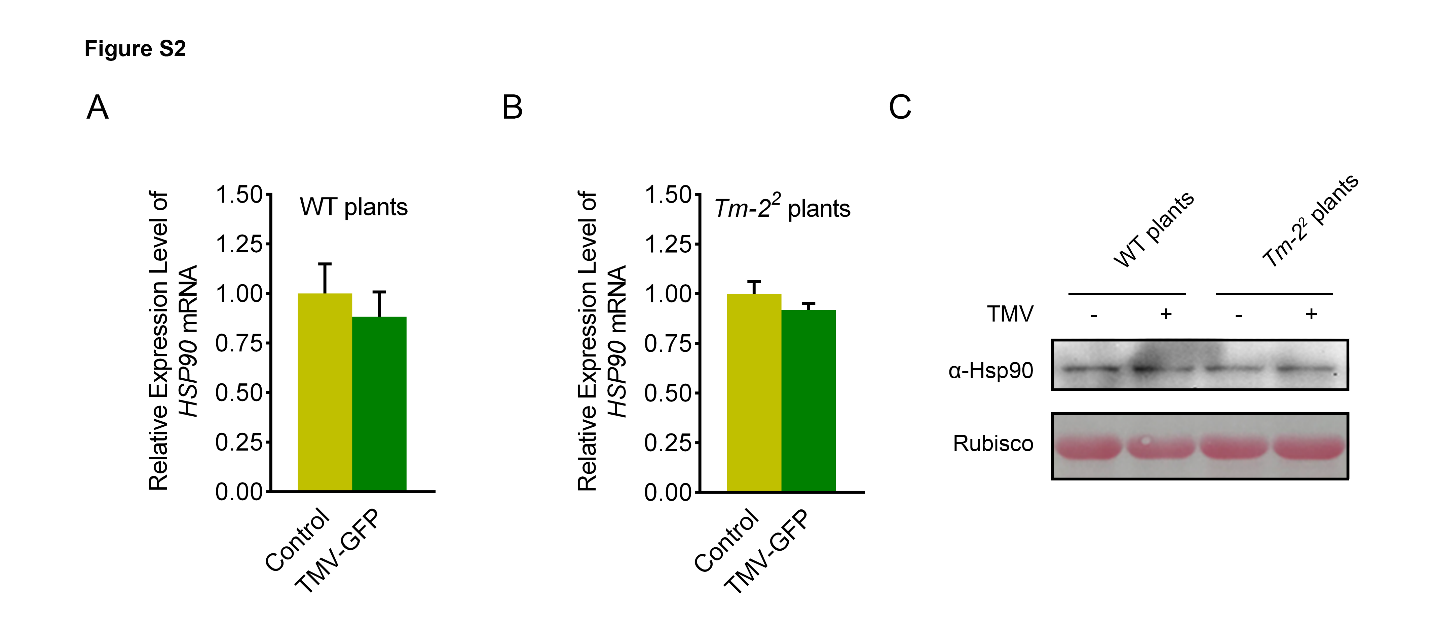


**Supplement Figure 2. *NbHsp90* expression and protein level were not induced by TMV infection and Tm-2^2^-mediate resistance.** **(A, B)** Real-time RT-PCR showed that TMV-GFP infection in wild type *N.benthamiana* (A) or *Tm-2^2^*-containing *N.benthamiana* (B) had no effect on *NbHsp90* mRNA level (means±SEM, n =3). *NbActin* mRNA levels were used as the internal control. **(C)** Wetern blot showed that TMV-GFP infection in wild type *N.benthamiana* (left) or *Tm-2^2^*-containing *N.benthamiana* (right) had no effect on NbHsp90 protein level. Equal loading of protein samples was validated by Ponceau Red staining of Rubisco subunit (lower panels).
